# A Community-Based Participatory Action Research with Women from Disadvantaged Populations: Strengths and Weaknesses of a Multiple Health Behaviour Change Intervention

**DOI:** 10.3390/ijerph19116830

**Published:** 2022-06-02

**Authors:** María Sanz-Remacha, Alberto Aibar, Ángel Abós, Eduardo Generelo-Lanaspa, Luis García-González

**Affiliations:** 1 Faculty of Health and Sport Sciences, University of Zaragoza, Plaza Universidad, 3, 50018 Huesca, Spain; msanzr@unizar.es (M.S.-R.); generelo@unizar.es (E.G.-L.); 2 Faculty of Human Sciences and Education, University of Zaragoza, Calle Valentín Caderera, 4, 22003 Huesca, Spain; aibar@unizar.es (A.A.); aabosc@unizar.es (Á.A.)

**Keywords:** disadvantaged population, Roma ethnic group, physical activity, participatory action research, healthy eating

## Abstract

Disadvantaged populations usually adopt risk behaviours, resulting in obesity and mental health-related disorders. Grounded in the socioecological model and self-determination theory, the aims were firstly to describe and implement a two-year multiple health behaviour change intervention, and secondly, to assess the strengths and weaknesses of the intervention. In total, 11 women from a disadvantaged population participated in this programme, which encompassed 117 sessions. Qualitative techniques were used to collect data and a thematic analysis was conducted. The variety of activities and the group-based intervention were the main strengths, and the decrease in attendance and the programme’s tight schedule were the main weaknesses. This is the first intervention in a disadvantaged population mainly comprised of Roma women. The design described in detail and its assessment provide relevant knowledge to improve their health status and decrease inequalities. The practical implications for future research are useful for replicating interventions in similar contexts.

## 1. Introduction

Disadvantaged populations, including the Roma ethnic group is one of the of the largest disadvantaged ethnic groups in Europe [1] In Spain, in particular, the Roma population is estimated to be around 850,000–900,000 people. This ethnic group is characterised by lower education levels, living in deprived neighbourhoods, having lower socioeconomic status, and having poorer health than non-Roma populations [2]. Roma women, especially, tend to have high overweight and obesity rates (i.e., 24.45% of Roma women have obesity in comparison to 10.22% of non-Roma women) [3,4], and more mental health disorders [4], which could be triggered by the prevalence of some risk behaviours (e.g., unhealthy diet and sedentary lifestyle) [5,6]), in addition to reporting low education levels, low socioeconomic levels [7,8], and living in deprived areas [9], are generally characterised by the adoption of health risk behaviour patterns (e.g., unhealthy diet and low physical activity (PA) levels) [5,10,11,12,13,14]. This type of population, especially women, displays higher overweight or obesity rates and poorer health status than advantaged populations, which could be emphasised by both a non-active lifestyle and an unhealthy eating pattern [15,16]. Given that their lifestyles are especially affected by contextual and social factors, such as perceiving more barriers to practise PA (e.g., family, economic, cultural barriers) [17] and experiencing a lack of social support to engage in PA [18], disadvantaged populations are less likely to successfully reach PA recommendations [15,19,20]. Regarding healthy eating, it is also known that disadvantaged populations usually follow diets that are low in fruit and vegetables [21,22]. Further, other educational factors, such as the lack of knowledge about nutritional information (e.g., caloric needs, serving sizes, or food nutritional values), may also trigger the prevalence of obesity in this type of population [23].

Considering the unfavourable conditions linked to women from disadvantaged populations to reach a healthy lifestyle, the design of intervention programmes aimed at promoting healthy behaviours in this type of population [24], and in particular in the Roma ethnic group [3], seems of paramount importance. In this sense, in addition to considering the population characteristics, needs, and barriers [17], previous multiple health behaviour change (MHBC) intervention studies have already identified some key characteristics that should be considered prior to designing an intervention programme aimed at improving healthy behaviours (e.g., PA and healthy eating) in disadvantaged populations [20,24,25,26,27] (e.g., low socioeconomic groups and immigrant families). There is a great amount of isolated evidence, but more evidence related to tailored interventions in specific ethnic groups (i.e., Roma ethnic group) is required. Likewise, identifying the strengths and weaknesses of the intervention programmes could be useful to put effective strategies in place, and to avoid implementing non-effective strategies in future programmes.

Firstly, the intervention should define the number of health-related behaviours to be modified in participants. Previous intervention studies in adults have reported improvements both after conducting single health behaviour change interventions (i.e., only one health behaviour is addressed, for example, PA), and multiple health behaviour change interventions (MHBC; i.e., at least two or more health behaviours are addressed, for example, PA and healthy eating) [28,29]. However, mixed and inconsistent results have been found in previous research with disadvantaged populations. Whereas single health behaviour change interventions (e.g., PA) have proven to be more effective than MHBC interventions [30], a recent review has shown greater improvements in healthy eating when interventions were based on two different health behaviours (e.g., PA and healthy eating) [24]. Despite these mixed results, the implementation of MHBC interventions aimed at improving health-related behaviours in disadvantaged populations is widely recommended because such interventions might be effective in terms of behavioural (e.g., improving health behaviours) or psychological improvements (e.g., improving well-being), and contextual or culture-related outcomes (e.g., cultural barriers to healthy lifestyles) [10,31]. Thus far, and to our knowledge, no MHBC interventions have been undertaken with the Roma population; so, considering the aforementioned positive consequences, this might be an optimal intervention design to improve health-related behaviours in this type of population.

Secondly, it is also important to determine the duration of the intervention programme. In this sense, the results from previous research on the optimal duration of interventions with disadvantaged populations have proven to be inconclusive. A recent umbrella review, aimed at analysing PA programmes in socioeconomically disadvantaged groups, found mixed evidence [20] while other reviews [11,25] found no differences in terms of the effectiveness with regard to the intervention duration. Nevertheless, short-term interventions (<6 months) might not be sufficient to achieve behavioural changes in community-based intervention programmes [32]; so, long-term interventions should be considered.

Thirdly, past research has focused on promoting health-related behaviours (i.e., PA participation and healthy eating) in disadvantaged populations by applying some behavioural and cognitive strategies. This is something that future programmes should consider [20,24,25,26,27,33]. For instance, in a qualitative review that focused on the effectiveness of PA- and diet-based interventions in low-income populations in the United Kingdom [25], different successful strategies were evoked, such as involving workers specialised in the participants’ culture or ethnicity, reporting wrong beliefs related to healthy behaviours promoted by mass media, improving participants’ self-esteem and self-control, or considering family support to change health-related behaviours. Likewise, another study conducted on disadvantaged populations suggested that performing PA in a non-evaluable context, and teaching how to train these skills, may be helpful to promote their autonomy in PA participation [34]. Regarding diet, a variety of effective healthy eating-related strategies (e.g., using the food pyramid, portion control sessions, setting short eating-related goals, providing discount cards to purchase vegetables and fruits) have been extensively included in interventions with disadvantaged populations [35,36,37,38]. In particular, one study with disadvantaged women, who were willing to modify their eating behaviours [39], demonstrated the effectiveness of different strategies, such as providing information about new and healthy recipes, suggesting the consumption of hypocaloric drinks, serving control, training in healthy eating habits, pointing out cheap food as a consumption alternative, or writing down daily consumed food.

Fourthly, the literature indicates inconclusive outcomes regarding the effectiveness of implementing theory- or non-theory-based interventions to promote health behaviours (e.g., PA and healthy eating) in disadvantaged populations [20,24]. However, the socioecological model and self-determination theory (SDT) have been suggested as optimal frameworks to develop health-related behaviour interventions in a variety of contexts (e.g., health domains), to promote the sustainability of PA interventions, and to involve health practitioners [40,41]. The socioecological model [41] provides support to MHBC interventions because it considers multiple levels of influence (i.e., intrapersonal, interpersonal, organisational, community, and public policy), allowing interrelations across them to be analysed. The comprehensive and practical approach of the socioecological model has proven to be a suitable framework for community-based interventions [42,43] in PA contexts [44] and with disadvantaged populations [45,46]. In addition, the socioecological model permits integration of other different theoretical frameworks [47], such as SDT. SDT [45] has been used to guide interventions in many and varied contexts, including PA settings [44], and with many disadvantaged populations [45,46]. In this sense, people tend to report positive results regarding self-determined motivation and the maintenance of healthier behaviour changes when their three basic psychological needs (BPNs; i.e., autonomy, competence, and relatedness) are satisfied [44,48]. Thus, the development of SDT-based strategies aimed at creating a need-supportive environment to fulfil the BPNs seems essential to reach optimal and more adaptive functional health outcomes (e.g., higher PA levels and healthier eating) [40,49].

Despite the increasing number of studies that address disadvantaged populations, applied research with the Roma population is still scarce, and, therefore, the conclusions related to the effectiveness of health-related interventions with this type of population are mixed, non-conclusive, and have very little detail [3,20,25]. Shedding more light on interventions aimed at promoting health-related behaviours in disadvantaged populations, especially in the Roma population, seems to be required to resolve this lack of knowledge [3,20]. This need becomes even more important with some specific ethnic groups that have well-defined cultural characteristics, such as the Roma population, a topic that has still not been examined in depth in the literature. Finally, and grounded in the socioecological model and SDT, this study aimed firstly, to describe the design and implementation of a two-year multiple health behaviour change intervention, and secondly, to assess the strengths and weaknesses of the intervention programme.

## 2. Materials and Methods

### 2.1. Study Design and Contextualisation

A community-based participatory action research (CbPAR) approach was used to undertake a health behaviour-based intervention programme named ‘Pio keeps moving’. CbPAR seems to be an optimal approach to carry out PA-related intervention programmes with disadvantaged populations, in particular with the Roma ethnic group [50]. This approach empowers participants; it helps to identify health inequities and address health promotion programmes in different ethnic groups [2,51]. CbPAR is a process in which the participants identify a problem and find solutions to solve it, promoting social transformation. This process requires multiple strategies, time, and in-depth information to understand the reality.

The ‘Pio keeps moving’ intervention was undertaken in a disadvantaged neighbourhood of Huesca, a mid-sized city located in the northeast of Spain. The neighbourhood, called Perpetuo Socorro, is located on the outskirts of the city. A total of 5998 people live there (11.43% of the population of Huesca), 23% of whom are immigrants and Roma. The intervention lasted for 20 months (from June 2016 to 2018), and it was structured around two main intervention topics: PA and healthy eating. Numerous public health practitioners, who work closely with the neighbourhood community (i.e., doctors, teachers, social workers, dietitians, and PA professionals), were involved in the design and implementation of the ‘Pio keeps moving’ intervention programme.

### 2.2. Participants and Recruitment

Fourteen women from disadvantaged populations in the deprived neighbourhood (details are omitted for peer review) signed a formal consent to participate in the study. Finally, a sample of 11 women (21.4% drop-out rate), representing a wide age range, from 27 to 58 years old (M = 37.72; SD = 8.34), participated in this 20-month intervention programme. In particular, seven participants (i.e., 63.6%) belonged to the Roma ethnic group. Although four of them did not belong to the Roma ethnic group, they had lived in the same neighbourhood for years, and they shared sociodemographic and cultural characteristics because of their daily coexistence in the same context. Hence, all participants shared the following common characteristics: (1) Residents in the (details are omitted for peer review) neighbourhood; (2) low incomes; (3) low education levels; (4) prolonged and continuous unemployment status; (5) receivers of government subsidies; (6) parents; (7) married, divorced, or widows; and (8) overweight or obese children diagnosed by the paediatrician (~85%) (see Table 1 for further information about participants’ sociodemographic characteristics).

One of the main characteristics of the CbPAR approach is to start the research based on a problem that has emerged in the community [33]. In this vein, both teachers from the elementary school and doctors from the hospital of the ‘Perpetuo Socorro’ neighbourhood detected a group of children (i.e., the participants’ children) with early overweight or obesity problems. The paediatrician transmitted this concern to the mothers who demanded a solution because they could not cope with the children’s problem. The paediatrician and teachers contacted other stakeholders, such as university researchers, community psychiatrists and dietitians, and the social worker who worked in the school neighbourhood, to find a solution. These stakeholders formed a work group, with the aim of tackling the obesity problem in children in the local community. Considering the mothers’ influence as one of the main determinants for children’s education [52], the social worker and the paediatrician acted as a gatekeepers and contacted women who had the following inclusion criteria to participate in the ‘Pio keeps moving’ intervention programme: (1) Have overweight or obese children (i.e., being the mother or legal tutor) who attended the neighbourhood school (details are omitted for peer review); (2) belong to a disadvantaged population; (3) have not finished secondary school education. Two representatives of the participants were also officially included in the work group following the CbPAR approach.

### 2.3. The ‘Pío Keeps Moving’ Intervention Programme

**Figure 1:**
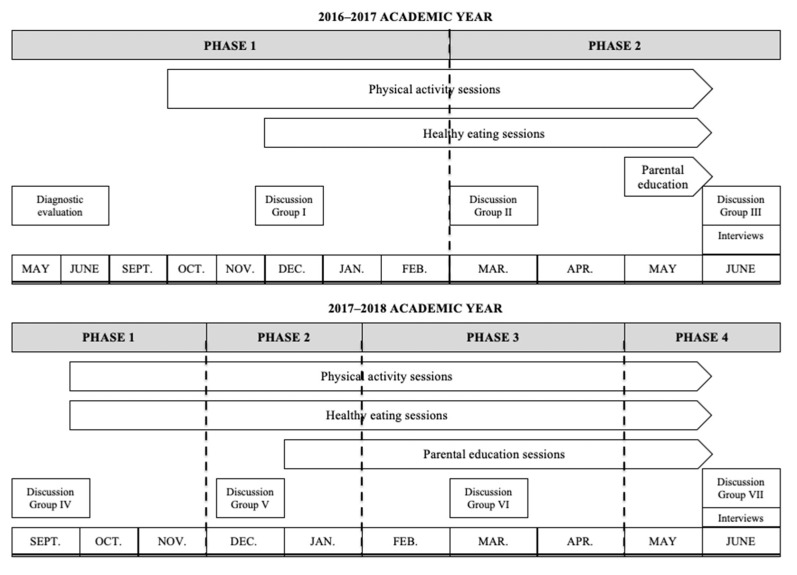
The timing of the ‘Pío keeps moving’ intervention programme.

‘Pío keeps moving’ is an MHBC intervention aimed at promoting two health-related behaviours in a group of disadvantaged women: PA and healthy eating, conferring special importance on PA behaviour. ‘Pío keeps moving’ was conducted over 20 months, and it was designed and implemented during the school year, given that mothers had more availability. The intervention was divided into four phases (see Figure 1). The first phase was called ‘Catching attention’, and its main purpose was to engage participants in the programme to acquire a healthy lifestyle. The second phase, ‘Awareness development’, aimed to increase participants’ awareness about the importance of incorporating healthy habits into their daily lives. The aim of the third phase, ‘Empowerment development’, was to improve self-adherence to an active and healthy lifestyle through the acquisition of different self-managing lifestyle resources. Finally, the fourth phase was named ‘Learning to be autonomous’, and its main aim was to facilitate autonomous decision-making associated with own health-related behaviours. Through this last phase, a pro-active behaviour in the participants was expected to be generated, which would facilitate self-management and the long-term maintenance of a healthy lifestyle. The first and second phases were briefly repeated (one month duration) at the beginning of the second academic year to avoid any possible problems in the adherence to the programme, and to recall some concepts after the summer break.

**Figure 2:**
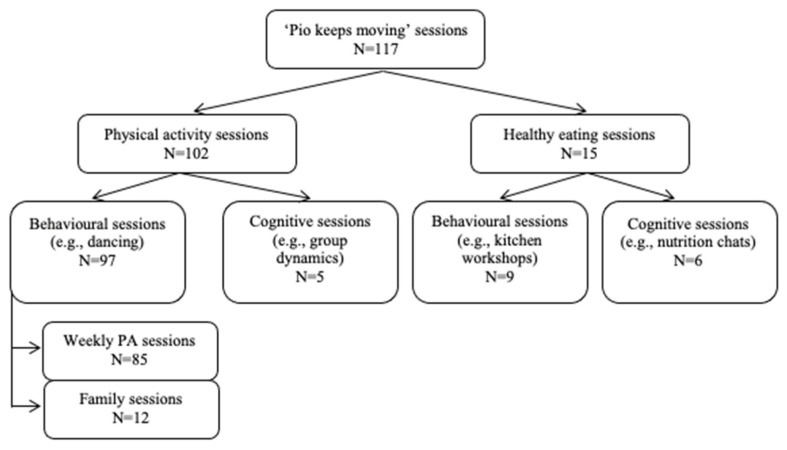
Type of physical activity and healthy eating sessions carried out in the ‘Pío keeps moving’ intervention programme.

The ‘Pío keeps moving’ intervention comprised a total of 117 sessions that were held throughout the four mentioned phases (see Figure 2). Following the CbPAR design’s characteristics, the number of sessions was not determined. Given that increasing PA levels was one of the main aims of the intervention, 80% of the sessions focused on modifying participants’ PA behaviour, whereas the rest of the sessions (i.e., 20%) focused on changing their healthy eating-related behaviour. As observed in Figure 2, the sessions aimed at changing each behaviour (i.e., PA participation and healthy eating) were, in turn, divided into practical and cognitive sessions. Practical sessions were designed so that women experienced the benefits of PA and healthy eating through practical tasks and exercises, whereas the aim of the cognitive sessions was to modify PA and healthy eating-related behaviours through the effect of metacognitive reflection and learning activities.

Three final sessions were organised at the end of the ‘Pío keeps moving’ intervention, the aim of which was for participants to maintain the behaviours they had learnt. These final sessions were a reminder of the importance of having a healthy lifestyle. At the same time, these three sessions were useful to show to participants a collection of all the activities and advice provided throughout the intervention (see Table 2 for further information).

Further, participants were provided with different and varied awareness-raising materials (e.g., thematic notebook) during the intervention programme. This type of material has been considered as an effective strategy to promote PA and decrease sedentary behaviour levels [42,53]. The thematic notebook, in particular, was used throughout the whole intervention programme to verify participants’ attendance. A personalised ‘Pío keeps moving’ stamp was used as a symbolic reward to give them positive feedback. Social networks (e.g., Facebook and WhatsApp) were also used to communicate with participants and to provide them with continuous motivation messages and positive feedback [54]. A private Facebook group was used to encourage participants and show them motivational videos aimed at promoting PA participation. A mean of two messages per week were uploaded in the private group. A WhatsApp group, with all the people involved, was not only useful to facilitate decision-making regarding the intervention programme but also to send them fast reminders about the scheduled activities before each session. The private Facebook profile and the WhatsApp group were managed by the lead researcher and the social worker, respectively.

#### 2.3.1. Physical Activity Sessions

A total of 102 PA sessions were performed over 20 months and each session lasted for around 1 h. Most of these PA sessions were ‘practical’ (i.e., 97), and they, in turn, were divided into two categories: group-based PA practical sessions (i.e., 85) and family PA-practical sessions (i.e., 12). Group-based PA practical sessions involved mothers and were conducted once or twice per week (depending on the moment of the programme). Family PA-practical sessions involved all the participants’ family members and were conducted twice per quarter, usually at weekends, lasting for around two hours. These types of family PA-practical sessions had proven effective in past health promotion interventions [55]. All activities performed in PA sessions are concisely reported in Table 1.

Both group-based and family PA-practical sessions were designed using SDT-based strategies to support the participants’ relatedness, competence, and autonomy needs (see Table 3). All need-supportive strategies were progressively applied to increase motivation for PA participation. Given the importance that relatedness support may have in promoting health-related behaviour changes [56], and with the aim of creating a friendly and trusting environment among participants, most of the need-supportive strategies applied during the first ‘Catching attention’ phase focused on the need for relatedness. During the second phase, ‘Awareness development’—not forgetting the use of the relatedness-supportive strategies—the majority of the strategies were especially focused on competence support to make participants feel more confident about PA practice. Finally, during the third phase, ‘Empowerment development’, and the fourth phase, ‘Learning to be autonomous’, the need-supportive strategies were essentially based on autonomy support so that participants could learn how to self-manage their own lifestyles. For instance, some of the issues tackled entailed making decisions about what kind of PA activities they would prefer, adequate PA intensity and frequency, or when and where they could practise these activities in their local context. In addition, special strategies were implemented at the end of the intervention to fully support autonomy. To illustrate this, the City Hall of Huesca offered participants the possibility of engaging in different organised PA sessions (e.g., aquagym, keep-fit exercises, relaxation activities, or bodybuilding activities) for free, during a limited period of time.

On the other hand, as cognitive tasks also seem be effective in increasing PA participation [57], the mothers were encouraged to reflect on the importance of adopting a more active lifestyle by means of five PA-cognitive sessions (i.e., one per quarter). Different dynamics were followed to reflect on PA (see Table 1 for further information).

#### 2.3.2. Healthy Eating Sessions

In addition to the 102 PA sessions, 15 practical and cognitive healthy eating sessions were carried out over a 20-month period (see Table 1). Each session lasted for around one hour. Nine were practical healthy eating sessions and comprised different types of activities. For example, activities such as workshops on how to do healthy shopping at the supermarket or how to make healthy recipes were carried out. All these workshops were given by dietitians and professional chefs. In addition, six cognitive healthy eating sessions were also performed. These cognitive sessions consisted of group dynamics (i.e., nutrition chats) based on common healthy eating-related topics that particularly concerned participants. The aim of these sessions was to provide them with knowledge to autonomously cope with eating-related problems by themselves (e.g., pictures related to portion size problems). Dietitians also provided advice based on feelings and hunger, and practical advice on how to control their eating anxiety in difficult stressful situations they had to deal with.

### 2.4. Data Collection

Qualitative techniques were used to collect information about the strengths and weakness of the intervention programme. Semi-structured interviews and discussion groups were conducted by the lead researcher in the school classrooms, a warm environment for the participants. Given the iterative reflective phases of the CbPAR approach, data was collected on a rolling basis [58]. All interviews and group discussions were audio recorded, and data collection took approximately 45 min. Semi-structured interviews were conducted in June of the years 2016, 2017, and 2018, respectively. Six discussion groups were conducted in December 2016 and 2017, March 2017, April 2018, and June 2017 and 2018, respectively. In addition, field notes were also used to obtain relevant information from the lead researcher’s perspective. Field notes were continuously collected after each session to triangulate data. Special attention was paid to participants’ feelings and the lead researcher’s thoughts about the development of the intervention programme. Four main statements guided this reflection process during the field note data collection: ‘How did the session go?’, ‘What were the lead researcher’s feelings regarding the participants?’, ‘Emerged needs during the session’, and ‘The intervention programme’s strengths and weakness from the researchers’ perspective’.

### 2.5. Data Analysis

The semi-structured interviews, discussion groups, and field notes were transcribed and analysed using NVivo Pro 11 software [59]. A preliminary qualitative data analysis was conducted. From a general point of view, an inductive thematic analysis with a semantic approach was used to assess the strengths and weaknesses of the ‘Pío keeps moving’ intervention programme. The lead researcher, a professional qualitative researcher, conducted the analysis, and then, the other researchers supervised the coding until agreement about the emerged themes, related to the programme’s strengths and weaknesses, was fully reached. This supervision process enhanced the quality of the analysis [60]. The coding data followed two specific research questions, resulting in two themes: the strengths and weaknesses of ‘Pio keeps moving’. The research questions were the following: ‘What strengths of the ‘Pio keeps moving’ programme would you highlight?’ and ‘What weaknesses of the ‘Pio keeps moving’ programme would you highlight?’.

## 3. Results and Discussion

Women from disadvantaged populations usually adopt unhealthy lifestyles, triggering detrimental health problems [5,11,13]. Consequently, numerous intervention studies, aimed at promoting health-related behaviours, have been conducted among different types of disadvantaged populations (e.g., low-income people, ethnic groups) [20,24,25,30], although the results and effects of these interventions are not entirely conclusive. However, to date, and to our knowledge, there are no particular intervention programmes based on the promotion of health-related behaviours in the Roma ethnic group, although recent studies have claimed the need to improve the Roma ethnic group’s health through health-related behaviour intervention programmes [3]. To overcome these concerns, the present study shows in detail the design of a 20-month MHBC intervention aimed at promoting a healthy lifestyle through the modification of two health-related behaviours (i.e., PA and healthy eating). Subsequently, an overall assessment based on the participants’ and researchers’ perceptions is reported. This evaluation will help to design and conduct higher-quality MHBC interventions with disadvantaged populations in the future.

### 3.1. Strengths of the Intervention Programme

The women identified several characteristics of the ‘Pio keeps moving’ intervention programme that may be highlighted as strengths. In line with past research conducted with participants with similar characteristics [25], the women pointed out the fact that staff members transmitted feelings of confidence, and provided regular and continuous positive feedback to all participants:

“Well, you’ve been very patient with us and you’ve been there for us. For example, you’ve not been discouraged when only a few people came; you have carried on and [told us], come on! Don’t worry, come on! You’ve fought”.

(Participant 5)

“…You’ve woken up the gymnasts we had inside, because if it were up to us, we restrict ourselves a lot”.

(Participant 7)

In this sense, it seems that the first strength of the ‘Pio keeps moving’ intervention could be the *staff’s adaptive personalities*. Therefore, considering that this type of population usually experiences low self-esteem and depression problems [3,61], the feelings of empathy, sensitivity, and affection from the staff should be considered as key characteristics of the programme that not only promote health-related behaviours but also improve the functioning of the intervention programme. Indeed, practitioners and researchers should provide feelings of confidence and regular positive feedback to all participants in this type of intervention.

If the staff’s adaptive personalities are pivotal, it seems just as important for participants to have sufficient opportunities to experience general positive feelings in the activities of the intervention programme. The data show how women had positive feelings such as enjoyment or confidence:

“For me, PA has been very enjoyable”.

(Participant 8)

“It’s given me more confidence in myself, and then, of course, the affection and companionship that we have established”.

(Participant 1)

In addition, participants claimed to be happy and confident during the intervention programme. These women also manifested an increase in self-confidence during the PA sessions, which could be associated with improvements in their perception of their competence. This may be explained by the progressive and well-thought implementation of need-supportive strategies during the intervention programme. Further, these positive feelings could also impact other areas of their personal lives such as satisfaction and well-being [45,62].

Likewise, participants also valued the large number of different activities performed both in the PA sessions (e.g., swimming, dancing, cycling) and healthy eating sessions (e.g., Mediterranean cooking workshop, international cooking workshop, shopping workshop). Participants and researchers indicated that ‘Pio keeps moving’ had strengths related to the *variety of activities*:

“The variety of activities we’ve done. Because, as I’ve said before, if it’s always the same, it’s boring; the time comes when you get bored. But, this year, there’ve been more activities than last year”.

(Participant 1)

This variety of exercises gave participants the opportunity to perceive their competence in the tasks performed. In this sense, the perception of competence has proven to be essential to continuing PA over the long term [63]. However, the variety of activities could also have triggered the experience of novelty in participants [64]. Recent research has proposed the need for novelty as a new BPN, which, if fuelled, may induce positive cognitive, behavioural, and affective consequences [64,65,66]. Thus, it seems that the variety of exercises could be considered the third important strength of the ‘Pio keeps moving’ intervention.

Further, the fact that it was a *group-based intervention programme* was perceived as a strength by participants and researchers throughout the whole intervention programme:

“Me, for example, well it’s the group that’s been formed; do you know what I mean? The friendship that’s been formed. That’s been great”.

(Participant 2)

In this sense, according to the social interaction hypothesis [67,68] and different systematic reviews [10,20], interpersonal relationships, which emerge among participants who take part in regular group-based activities, may help to cope with physical and mental health problems by creating wider social networks, avoiding feelings of loneliness, and increasing personal resources [69]. Hence, the fourth fundamental strength of the ‘Pio keeps moving’ intervention was the group-based sessions and continuous support of the participants’ relatedness through SDT-based strategies. This strength could be especially significant in disadvantaged populations who often suffer from important self-esteem problems [3,15].

In addition to the aforementioned participants’ perceptions, the researchers also specifically perceived other strengths of the ‘Pio keeps moving’ intervention programme. Firstly, an important feature of the programme was the *long duration of the intervention programme* (20 months). Recently, studies have found that long-term interventions have shown improvements in the second year of the intervention programme [70]; so, long-term intervention programmes facilitate not only the acquisition but also the maintenance of health-related behaviours [40]. This long-term planning seems especially important in disadvantaged populations (notably in the Roma ethnic group) due to their poor levels of health, and might require more time to experience changes and integrate new health-related behaviours. Moreover, after the intervention, the results of the ‘Pio keeps moving’ intervention showed that some of the behaviours needed more time to be changed [65]. Secondly, researchers also perceived the proposal of a *schedule and flexibility in terms of activities and organisation* as another strength. Family, labour, and economic barriers to participation in PA are usually experienced by disadvantaged populations [17]. Consequently, the schedule was continuously modified according to the participants’ responsibilities and priorities (e.g., days of the week for the sessions, start time). This characteristic seems important to improve success rates in an intervention programme with this type of population. Thirdly, another perceived strength was *the simultaneous intervention in two health-related behaviours* (i.e., PA participation and healthy eating). Although there are inconclusive findings about the effectiveness of working simultaneously or sequentially on health-related behaviours, given that disadvantaged populations need to modify many of their health-related behaviours, it could be assumed that intervening simultaneously would impact their PA levels and that their eating habits might be more effective as these trigger a summative effect. Fourthly, according to the above strength, *the inclusion of different public health practitioners* in the intervention programme was also fundamental. Likewise, the practitioners should know the target population and have previous experience working with them [71], as in this way, their advice will focus on the same objectives and goals, which become more numerous and effective. Finally, we would also like to highlight the *participation of external organisations* (e.g., YMCA, University, City Hall) as a strength of the intervention programme. The collaboration of these external institutions was essential to financing some activities and facilitating access to material resources or sports facilities.

### 3.2. Weaknesses of the Intervention Programme

Participants and researchers also identified several weaknesses of the intervention programme. For example, one of the weaknesses of ‘Pio keeps moving’ was the *decrease in attendance at the end of the second year:*

“The fact that they’ve not attended the last few days, and I didn’t know [they hadn’t come]. Everything else has been fine”.

(Participant 7)

“That the group has disappeared”.

(Participant 1)

Participants perceived that the group was starting to break up due to the decreasing attendance at the end of the intervention programme. Low participation is usually common in disadvantaged populations [20]. In this sense, health-related behaviour interventions should implement strategies to create warm relationships among participants and maintain regular high rates of adherence. To do so, future interventions should pay even more attention to need-supportive strategies focused on relatedness throughout the whole intervention programme, and social support from family and friends, which has been considered a key factor for encouraging PA in disadvantaged populations [20,24,72].

Another weakness perceived by participants and researchers was related to *the tight schedule of the intervention programme*:

“The schedule, because, for example, when I sorted things out to be able to come at midday, it wasn’t possible, because it was held in the mornings. And then, it couldn’t be changed because of those who came in the mornings; but of course, they’re not going to change just for me, for one person. The rest was OK (…) The schedule”.

(Participant 5)

“More negative points… I don’t know… the schedule… I don’t know”.

(Participant 7)

The schedule became an important problem during the second year. Most of the participants were responsible for looking after their family, and some of them had short-term contract jobs, and they had to face constantly changing work schedules. Recent studies [17,73] with disadvantaged populations have reported the perception of a lack of time as a barrier to participating in PA, which could apply in the cases of having this type of changing schedule. To cope with this problem, health promotion-related interventions should offer more attendance opportunities by doubling sessions, such as creating morning and afternoon groups with common work schedules and offering PA sessions at nearby places (e.g., at the neighbourhood sports centre). Participants could choose the schedule that suited them best according to their personal characteristics.

In addition to the participants’ perceptions, some weaknesses of the ‘Pio keeps moving’ intervention were also perceived by the researchers. Firstly, a *huge number of weekly sessions* was offered by the intervention programme during specific periods (e.g., the last term in the first year). Too many sessions in a limited period of time could have a negative influence in different ways: (1) Increasing dropout rates of participants with low attendance, who could leave the programme; (2) women with regular attendance could drop out of the programme because they feel that they are relegating their ‘true’ responsibilities; and (3) women might drop out of the programme and join different, less time-demanding, sports activities, instead of ‘Pio keeps moving’. Secondly, in line with recent studies that show the importance of considering the ethnicity of professionals that lead intervention programmes [56], another weakness perceived was the *absence of practitioners belonging to the same ethnic group or with the same characteristics as the participants*. The presence of a person of reference from the same ethnic group could encourage participants to modify their unhealthy behaviours due to both the message they portray and as role models. Nevertheless, to facilitate closeness between the participants and the members of staff, the ‘Pio keeps moving’ intervention tried to ensure that the people involved in designing the programme were mostly women. Finally, the researchers also identified that *participants were excessively dependent on some of the health practitioners*, in particular on the social worker. Despite a recent systematic review, which showed that regular communication between participants and public health practitioners induced higher effectiveness [20], many human, material, and temporal resources are required to avoid excessive dependence on one person.

In the present study, several limitations should be considered. A purposive and small sample was used due to the problems of accessibility in this population, and the low rate of voluntary participation, despite the use of gatekeepers (i.e., social worker and paediatrician). However, the small sample is coherent with the methodology used, which requires a small size to obtain detailed data. In addition, our study exclusively comprised a sample of women, which reflects the difficulty in accessing a male sample, and the influence of the cultural context in this type of population. Further research with large male samples of this ethnic group is required to achieve a tailored intervention. Finally, an analysis of the differences perceived by Roma and non-Roma people related to health-related behaviours could be useful to improve their health status.

## 4. Practical Implications for Future Interventions in Disadvantaged Populations

According to the strengths and weaknesses of the ‘Pio keeps moving’ intervention programme, and considering the behavioural and motivational changes perceived by the participants after ‘Pio keeps moving’s implementation [65,66], some practical implications should be considered to address future MHBC interventions in disadvantaged populations. Firstly, long-term interventions seem to be required to provide sufficient time to change unhealthy behaviours in disadvantaged populations. Nevertheless, simultaneously addressing behaviours throughout MHBC programmes may provide good results in disadvantaged populations. Secondly, the adaptive personality and the ethnicity of the practitioners are two characteristics that, when possible, should be considered (e.g., involving confident practitioners that belong to the same ethnic group as the participants) to ensure proper development of the intervention programme (i.e., high participation, effectiveness). Furthermore, it is also important for the public health practitioners involved in the programme to come from different health areas (i.e., dietitians, doctors, PA professionals), and it is even better if they have previous experience with the target population. Thirdly, it is highly recommended that group-based interventions are performed and the participants’ need for relatedness is supported. Fourthly, it seems important to offer participants varied and new activities. Likewise, it is necessary that demanded activities are selcted that adapt to the participants’ characteristics and needs. Fifthly, the intervention programme should offer participants different attendance possibilities (e.g., morning and afternoon schedule). This session flexibility may help to cope with the low attendance, previously identified in past research as a common and strong barrier to PA in disadvantaged populations. Finally, it is important for future replications of the ‘Pio keeps moving’ intervention programme to also consider aspects of the target population (i.e., culture, needs, interests, barriers) to adapt the intervention to the participants’ specific demands and context.

## 5. Conclusions

This research shows that a CbPAR approach could be a useful design for promoting health-related behaviours in women from disadvantaged populations, especially Roma women. The results of the present study are supported by other research [65], which showed the behavioural changes perceived by this disadvantaged population after the ‘Pio keeps moving’ implementation. The phases and timeline developed throughout the programme are accurately described so they can be considered for future interventions. The implemented SDT-based strategies were shown and described in this study, and undertaking similar interventions to promote a healthy lifestyle in disadvantaged populations might be effective. Similarly, the strengths perceived by researchers and participants contribute to an increased knowledge about what strategies and activities could work in this type of population. In contrast, the perceived weaknesses should be avoided or modified as much as possible, depending on the target population and the context. However, more studies aimed at promoting healthy lifestyles in the Roma ethnic group and disadvantaged populations are required. All this detailed information, together with the practical implications for future research, is expected to be useful for researchers and practitioners to implement other interventions in similar contexts. To sum up, this research is only a first step in the literature, and more interventions are necessary to increase practical evidence on how to improve health-related behaviours in disadvantaged populations, especially in the Roma population.

## Figures and Tables

**Table 1:** Participants’ sociodemographic characteristics.

ID	Age	Marital Status	Ethnic Group	Employment Contract
1	42	Married	Non-Roma	Short-term contract
2	32	Married	Roma	Unemployment
3	36	Widow	Roma	Short-term contract
4	58	Divorced	Non-Roma	Short-term contract
5	44	Widow	Roma	Unemployment
6	38	Married	Roma	Short-term contract
7	39	Married	Roma	Unemployment
8	31	Married	Non-Roma	Short-term contract
9	29	Married	Non-Roma	Short-term contract
10	40	Married	Roma	Unemployment
11	28	Married	Roma	Unemployment

**Table 2:** Specific actions designed for the ‘Pío keeps moving’ intervention.

		Practical Sessions		Cognitive Sessions	Awareness Actions
Behaviours	Academic Year	Group-Based Sessions	Family Sessions		
PA	2016–2017	Dancing different music styles (1,2). Swimming for beginners (2). Circuits of muscle-building and toning exercises with elastic bands, and alternative material (e.g., bosu, suspension training) (1,2). Indoor cycling sessions (2). Back pain prevention sessions (2). Walking in the area around Huesca (e.g., parks, paths) (2,3,4).	Childish game party: ‘Pirate party’ (1). Aquatic party for children (1): ‘Hawaiian party’. Family walking (4.5 km) in the area around Huesca. Traditional games (2). Game of clue (2).	The main topics of the sessions were: What kind of activities could not you do before and currently you can? (1). Reflections about PA recommendation levels (frequency and intensity) (2). The intensity of daily activities (2). Time management strategies: C30/D30 based on encouraging to participants to ‘take 30 min of PA and 30 min of sedentary activity’ (2). Analysis of their accelerometer data (a comparison between years) (2).	Four personalised notebooks with the following topics: Benefits of PA participation. Stretching exercises. C30/D30 strategies related to PA recommendations. Activities realised in PA cognitive sessions (e.g., activities that they are able to perform and they are not able to perform). Activities of different intensity. Short-term aims. PA activities offered by the City Hall. All notebooks have spaces to write their feelings and mood during activities.
	2017–2018	Dancing different music styles (1,2,3). Tennis games (2). Swimming for beginners (2,3). Circuits of muscle-building and toning exercises with elastic bands, and alternative material (e.g., bosu, suspension training). (2,3,4). Spinning (2,3,4). Back pain prevention sessions (2, 3). Walking in the area around Huesca (e.g., parks, paths) (1,2,3,4).	Aquatic party for children (2). San Jorge’s walking: popular walking in the city of Huesca with relatives (3). Game of clue (3,4). Kin-ball (3). The board game called ‘Pío keeps moving’ to finish the programme (4)	Let us describe: how do we see ourselves in PA practice? (interactive and manipulative dynamics to define themselves) (1). Motivational videos about PA activities that they do (1). PA recommendation levels (frequency and intensity) (1). What kinds of activities could you not perform before and currently you can? (2). Intensity of the daily activities (3). Barriers toward PA: limitations perceived by the participants toward PA (3).	
HEALTHY EATING	2016–2017	Active healthy shopping following a healthy shopping list worked on in previous sessions (2). Kitchen workshop: Mediterranean healthy recipes and international healthy recipes. All recipes were described with ingredients’ prices, amounts, and healthy rations (2).		Nutritional pyramid (1). Nutritional values (e.g., calorie intake linked with PA and eating) (1). Analysing own receipts (e.g., they send pictures of meals cooked by them) (2). Healthy receipts and alternatives (2). Ration sizes (2). What kinds of foods should they eat? Why? (2).	Four personalised notebooks with the following topics: Nutritional pyramid. Importance of healthy eating, what types of food should be avoided. Healthy recipes. Pictures about added sugar intake in each food.
	2017–2018	Kitchen workshop: Mediterranean healthy recipes and international healthy recipes. Recipes contained the ingredients’ prices, amounts, and rations (3).		Barriers toward healthy eating: limitations perceived by the participants toward healthy eating (3). Nutritional values (e.g., calorie intake linked with PA and eating) (2,4). Added sugar intake (3).	
FINAL SESSIONS	2017–2018(4)	Aim of ending sessions: to close the program and remove consciousness to promote sustainability of the behavioural change First session (4): Projection of a video with a set of the interventions’ photos: questions about the video (4) - What feelings and memories have been transmitted when you have seen the video? - What moments would you like to repeat? - What experiences would you like to repeat? What experiences would not you like to repeat? Why? - Has ‘Pío keeps moving’ achieved your expectations? - Do you believe that have you made a high effort for 2 years? - After 2 years, how would you describe yourself regarding PA practice? They should compare their description with the description made during the program. Second session (4): - ‘Photovoice’: set of photos taken during the sessions. Associated question: From activities practised during the program, what did you think you would not be able to do? How do you feel after? - Dynamic activity: What food activities have you practised? What did you learn? - Healthy receipts: What receipts have you cooked in your home? What problems have you encountered? How did you solve them? What is the family’s opinion about these receipts? - A reminder of the nutritional pyramid - Challenges encountered: linked with the problems transmitted by them (e.g., sugary drinks, amounts, added sugars, fast food, etc.) Third session (4): - PA activities offered in the city that are compliant with their characteristics (e.g., cheap activities, different timetables, variety of activities depending on the subject’s level, etc.).			

The numbers between brackets describes the programme’s phase (i.e., 1: Phase 1 ’Catchin attention’; 2: Phase 2 ‘Awareness development’; 3: Phase 3 ‘Empowerment development’; 4: Phase 4 ‘Learning to be autonomous’. Each behaviour (i.e., PA and healthy eating) is divided into two academic years.

**Table 3:** Basic psychological needs supportive strategies designed for the ‘Pío keeps moving’ intervention.

Strategies’ Topic	Relatedness-Supportive Strategies
‘Pío keeps moving’ decisions	Decisions about the organisational-intervention programme were discussed in groups. For example, the types of activities, timetables, and frequency of the sessions.
Sizes of groups	Continuous change in the size of groups carried out in the different PA sessions. For example, different couples.
Cooperative games	Teamwork and dynamic activities aimed to obtain an agreed solution.
Environment	Participants chose a friendly and comfortable environment to perform the activities in.
Mood’s PA professional	The PA professional was a trusted person, who was empathetic and patient throughout the intervention.
Funny meetings	Having dinner at the end of the first year; healthy snacks or coffee meetings to talk about the programme and share the participants’ experiences.
Social networking	Creating a WhatsApp group and Facebook profile for participants to keep in touch, and using it to remind participants about the timetables and meeting to perform PA. The motivational videos and positive feedback were provided by the Facebook profile.
Competence-supportive strategies	
Initial information	Participants were informed about the activities at the beginning of the sessions by the PA professional.
Variety of material and places	Participants tried out different type of new materials (e.g., elastic bands, bosus, TRX, fitballs, medicine balls) and places during the intervention (e.g., fitness centre, indoor sports centre facilities, swimming pool).
Variety of activities	A variety of group activities. For example, walking in groups around the city, familiar activities on the weekend, dancing in groups, circuits of muscle-building and toning exercises.
Adapted activities	PA professional offered the intensity and frequency levels in each activity depending on the subject. In addition, the trainer/staff designed activities for people who had suffered some type of injury.
Number of activities	Whenever possible, two or more different exercises were carried out in each PA session, and a vast body of opportunities were offered to achieve success.
Improvement feedback	Several strategies were implemented as follows: Providing positive feedback on the participants’ PA levels, practising activities that they could not perform and they could perform (e.g., at the beginning, they walked 3 km and at the end, they walked 5 km). Participants received individual and group positive feedback before, during, and after the PA sessions to encourage them and emphasise their PA improvements.
Motivational videos	Recording videos during the sessions (e.g., dances). From them, participants could analyse themselves and show them and compare their improvement.
Goals	Several strategies were implemented as follows: Participants were informed about the session’s goals and activity tasks at the beginning of the session. At the beginning of each academic year of the intervention, short-term goals and long-term goals were set out. Each participant set out individual and group goals in each session.
Autonomy-supportive strategies	
Making decisions	Several strategies were implemented as follows: Break time during PA sessions when they felt tired. The exercise intensity. The type of exercises, activities, and materials. The order of the exercises (e.g., first dancing, and later, circuits of muscle-building and toning exercises). The representative logo for ‘Pío keeps moving’ and the t-shirts’ style (colour). The music (songs) and dance steps. The path during walking activities around the city of Huesca.
PA events	Participants were encouraged to participate in PA events (e.g., popular walking) carried out in the city of Huesca (Spain) throughout the intervention.
Autonomous PA	Participants were encouraged and empowered to participate in autonomous PA. PA professional provided information related to friendly environments in the city to perform PA, material (e.g., PA notebook), City Hall’s activities with reduced prices (e.g., relaxation, gymnastics maintenance).
